# Late-Onset Metastasis of Renal Cell Carcinoma into a Hot Thyroid Nodule: An Uncommon Finding Not to Be Overlooked

**DOI:** 10.1155/2015/268714

**Published:** 2015-01-05

**Authors:** Luca Foppiani, Michela Massollo, Patrizia Del Monte, Roberto Bandelloni, Anselmo Arlandini, Arnoldo Piccardo

**Affiliations:** ^1^Internal Medicine, Galliera Hospital, Mura delle Cappuccine 14, 16128 Genoa, Italy; ^2^Nuclear Medicine, Galliera Hospital, Genoa, Italy; ^3^Endocrinology, Galliera Hospital, Genoa, Italy; ^4^Unit of Pathology, Galliera Hospital, Genoa, Italy; ^5^Surgery, Galliera Hospital, Genoa, Italy

## Abstract

We report the case of a 74-year-old man with a four-year history of right nephrectomy for clear cell renal carcinoma (CCRC) who was diagnosed with hyperthyroidism. On ultrasound (US), a 5 cm solid isohypoechoic nodule with intranodular vascularization was found in the left thyroid lobe. The nodule was deemed autonomous on ^99m^Tc thyroid scan. Methimazole was started and serum thyroid hormone levels quickly normalized; euthyroidism was maintained with a very low dosage of antithyroid drug. Over time, compressive symptoms and local pain occurred and US revealed growth of the nodule. Total thyroidectomy was performed and the combined histological and immunohistochemical evaluation deemed the nodule compatible with metastasis of CCRC; on 2-year follow-up, no tumor relapse was ascertained. In patients with a history of cancer, a thyroid nodule, even if hyperfunctioning, must be suspected of being a metastasis and investigated. Hot nodules, which are largely benign, may be vulnerable to metastatic colonization owing to their rich vascularization. In these cases, surgery may be curative.

## 1. Introduction

The presentation of a thyroid nodule years after the treatment of a primary cancer often raises a diagnostic dilemma. Until the early 2000s, metastases to the thyroid were deemed mostly asymptomatic and an infrequent cause of clinical problems [1, 2]. Reported cases of secondary thyroid cancer requiring surgery were therefore few [3]. This notion has recently been challenged in a literature review of over 300 patients, which points out that metastases to the thyroid gland, besides not being clinically uncommon, are often symptomatic [4]. CCRC is the most common primary tumor which metastasizes to the thyroid [2, 4]; moreover, it diffuses in an unpredictable manner to organs and can show very late recurrence (up to 20 years). In 30% of cases, metastases are present on diagnosis. This hypervascularized tumor is associated with numerous arteriovenous shunts, whereby hemodynamic factors play an important role in the seeding and subsequent growth of metastases through the vascular route [5]. In clinical practice, ultrasound features are unable to distinguish primary from secondary thyroid tumors. Thus, cytological and, mostly, combined histopathological and immunohistochemical analyses are required for a proper diagnosis [4]. In selected patients with a solitary thyroid metastasis of CCRC, surgery may significantly increase survival [6]. Here we report the case of a patient operated on 4 years earlier for CCRC, who developed a large thyroid metastasis of the primary tumor, which was unexpectedly detected within a hyperfunctioning nodule.

## 2. Case Report

A 74-year-old man underwent medical examination for palpitations. The patient's history documented a right nephrectomy for a clear cell renal carcinoma (CCRC) (pT2G2N0) 4 years earlier. No relapse on CT was reported during follow-up.

**Figure 1:**
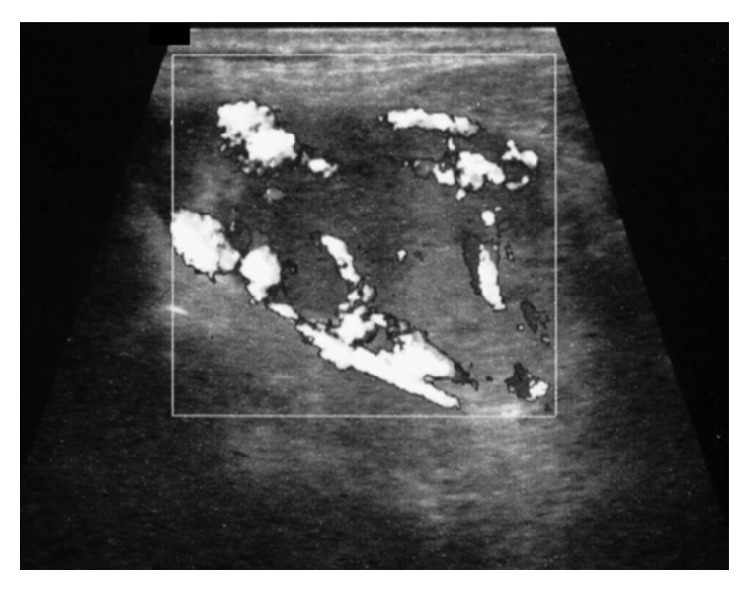
Thyroid ultrasonography showing a left 5 cm solid and isohypoechoic nodule with peripheral and intranodular vascularization.

**Figure 2:**
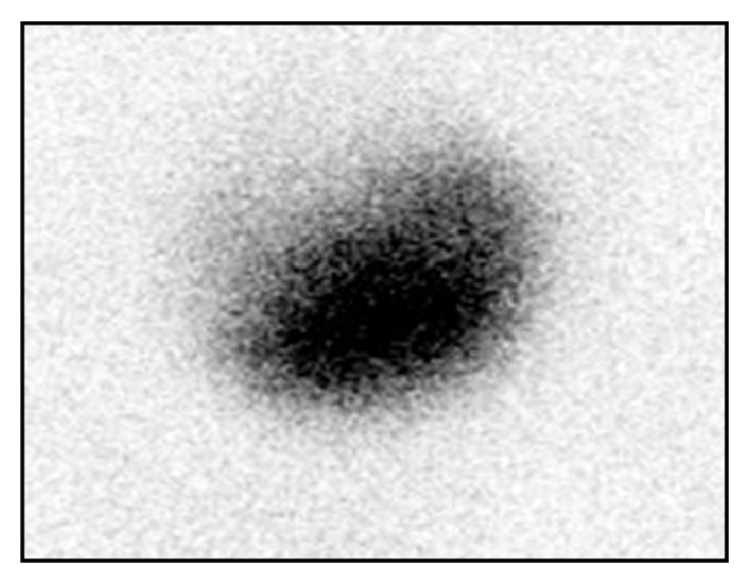
^99m^TC thyroid scan showing exclusive tracer uptake in the left nodule with inhibition of the surrounding parenchyma.

**Figure 3:**
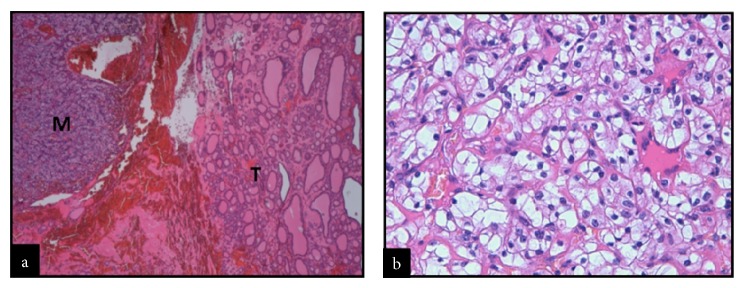
Representative histological (hematoxylin-eosin stain, 40x) micrographs showing normal thyroid tissue (T) and metastatic tissue (M) (a). A greater (400x) magnification of the metastatic tissue, featuring large cells arranged in nests and cords with optically clear cytoplasm and enlarged nuclei displaying coarse chromatin, is shown in section (b).

**Figure 4:**
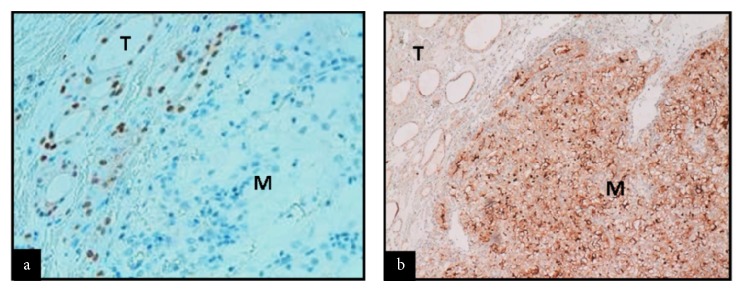
Thyroid transcription factor-1 (TTF-1) immunohistochemistry (100x) shows nuclear expression in the thyroid tissue (T) but not in the metastatic tissue (M) (a) and CD10 expression (40x) in the cell membrane of the metastatic tissue (M) but not in the thyroid tissue (T) (b).

Chemistry was unremarkable, whereas thyroid function assessment showed hyperthyroidism with prevalent FT3 secretion (FT3: 8.0 pg/mL, n.v. 1.8–4.6, FT4: 1.85 ng/dL, n.v. 0.9–1.7, TSH <0.005 *μ*IU/mL, n.v. 0.3–4.2). On physical examination, a large painless mass was palpable in the left thyroid lobe. Thyroid ultrasonography (US) revealed a 5 cm solid, isohypoechoic nodule with intranodular vascularization (Figure 1) in the left thyroid lobe. ^99m^TC thyroid scan (111 MBq) showed exclusive tracer uptake in the nodule, with inhibition of the surrounding parenchyma (Figure 2). Thyroid autoantibodies proved negative and calcitonin levels were normal. Methimazole 5 mg twice a day was started; mild hypothyroidism (TSH: 3.9 *μ*IU/mL, FT4: 0.7 ng/dL) was soon obtained and the antithyroid drug was gradually tapered to a very low dosage (2.5 mg/day), which maintained euthyroidism over time. Given the size of the nodule, surgery was advised but was refused by the patient because he was asymptomatic. Radiometabolic therapy was similarly declined. Fine needle aspiration biopsy (FNAB) proved inadequate because of scant cellularity (THY1), but the patient refused to repeat the procedure. One year after the first evaluation, the patient started complaining of compressive symptoms and local pain. Thyroid US revealed that the nodule had grown (maximum diameter 6 cm). Total thyroidectomy was performed and histopathology showed a neoplastic nodule composed of both typical nodular goiter and large cells arranged in nests and cords with optically clear cytoplasm and enlarged nuclei displaying coarse chromatin (Figures 3(a) and 3(b)). Immunohistochemistry proved negative for specific thyroid transcription factor-1 (TTF-1) (Figure 4(a)) and thyroglobulin (not shown) and positive for CD10 (Figure 4(b)). These features, together with the patient's history, were consistent with a metastasis from CCRC. L-Thyroxine replacement therapy was started and euthyroidism was achieved and maintained. On 2-year follow-up, no evidence of recurrent renal carcinoma was found on whole-body CT, and US did not show neck relapse.

## 3. Discussion

The incidence of metastases to the thyroid gland ranges from 1% to 24% of malignant tumors in autopsy series [2, 4] and in 1.4–3% of all patients who undergo surgery for malignancy in the thyroid gland [4].

In contrast with what was previously accepted [1], a recent review of literature data from the years 2000 to 2010 on 372 patients [4] demonstrated that metastases to the thyroid often (two-thirds of patients) present with clinically disturbing symptoms, such as neck swelling, dysphagia, and dysphonia, and, more rarely, with respiratory distress requiring urgent thyroidectomy or tracheostomy. In that review, nearly half (44.2%) of metastases to the thyroid gland occurred in glands with primary benign or malignant tumors or autoimmune thyroiditis.

Further literature data suggest that thyroid tissue that is deranged as a result of goiter, primary tumor, or thyroiditis may be more susceptible to metastasis owing to its decreased oxygen and iodine content, though others have not confirmed these findings [7, 8].

We report a case of CCRC which unusually metastasized into a hot nodule of the thyroid gland 4 years after nephrectomy. CCRC is one of the tumors most prone to metastasize to the thyroid and accounts for 12–34% of all secondary thyroid tumors [4, 9]. Metastases may be the first manifestation of CCRC or a synchronous, or more frequently metachronous, metastasis of a known CCRC [4, 7].

The recent literature review by Chung et al. [4] of 372 cases, 180 of which (48%) with thyroid metastasis from CCRC, shows that metastases to the thyroid gland are solitary in up to 40% of patients. Other reports on smaller numbers of patients confirm that metastases from CCRC are mostly solitary [10].

A very recent review of all thyroid FNABs performed at the Mayo Clinic from 1980 to 2010 identified 97 patients with a metastatic tumor of the thyroid gland and confirmed that the most frequent source of metastasis was the kidney (22%) and that these tumors displayed the longest time from diagnosis to metastasis (mean 113 months, range 3 months to 20 years). It also addressed various important points: (i) overall, the resected metastatic thyroid lesions were mostly solitary; (ii) patients surviving ≥ 3 years after the discovery of metastatic disease of the thyroid mostly (37%) had CCRC; (iii) thyroidectomy (performed in 41 out of 91 patients) resulted in improved median survival (30 months) in comparison with patients without surgery (20 months), but this did not reach statistical significance, possibly owing to the rather low number of subjects included [11]. By contrast, a report on 36 patients with thyroid metastases from different primary tumors pointed out that thyroidectomy did not increase patients' survival [12].

On the whole, in patients with plurimetastatic disease, thyroidectomy should be performed only to palliate compressive symptoms [7].

Although several reports of renal carcinoma metastasis to the thyroid exist [4, 11], to the best of our knowledge only one other case of CCRC metastasizing within a hot thyroid nodule has been previously described as letter to the editor, in the context of a study addressing the prevalence of malignancy in a cohort of patients with uni- or multinodular toxic goiter [13]; in that report, however, the patient's follow-up was not available, the time to tumor recurrence was longer, and immunohistochemical studies were not accurately described as in the present report.

In our patient, clinical and US features of the thyroid nodule failed to lead to a correct diagnosis, and FNAB did not yield a sufficient number of cells to be analyzed. In this regard, a recent large review found that thyroid FNAB cytology may fail to yield a correct diagnosis of metastatic CCRC in nearly 30% of patients [4].

In our patient, the CCRC metastasis to the thyroid occurred 48 months after nephrectomy, an interval shorter than those reported in the literature (68–130 months) [4, 11]. This finding is probably related to the fact that our patient searched medical attention for symptoms eventually related to hyperthyroidism due to toxic nodule and thus hormonal and instrumental work-up was carried out.

Apart from the fact that clinical and US features are aspecific, it may also be a difficult task to distinguish between primary (mostly anaplastic carcinoma or the unusual clear cell variant of follicular carcinoma) and metastatic thyroid tumors on the basis of cytology or histology.

In this regard, immunohistochemistry for thyroglobulin (which may, however, be negative in anaplastic thyroid carcinoma) and TTF-1 (more specific) is very useful in distinguishing primary (positive) from secondary (negative) thyroid tumors [7]. In the case reported, a combined histological and immunohistochemical evaluation (negativity for thyroglobulin and TTF-1 and positivity for CD10), together with the patient's history, led to the diagnosis of metastasis from CCRC.

The peculiar feature of our case is that the solitary CCRC metastasis developed within a toxic nodule; a possible explanation for this finding is that a preexisting hyperfunctioning nodule might be more vulnerable to tumor colonization on account of its hypervascularity, which may constitute a route for the seeding of cancerous cells. Most thyroid metastases do not usually affect thyroid function [4, 11]. However, in our patient, the hyperfunctioning thyroid nodule was probably gradually replaced by metastatic tissue, which may have caused the progressive reduction of its hypersecretory activity. This would explain the need to reduce thyrostatic therapy to a very low dosage, which probably would otherwise have been insufficient to control hypersecretion by such a large hot nodule.

In our patient, total thyroidectomy was performed owing to compressive symptoms, which, as recently reported in the literature [4, 11], are quite common in thyroid metastases. Clinical and instrumental follow-up proved negative for tumor relapse after 2 years.

## 4. Conclusion

In patients with thyroid nodule/s and a history of malignant disease, relapse or the progression of malignancy within the thyroid must be considered until proven otherwise. Furthermore, we suggest considering the rare but possible presence of metastasis in the context of hot thyroid nodule and a medical history of CCRC. In selected cases, surgery can be curative.
